# Review of the Risks of Some Canine Zoonoses from Free-Roaming Dogs in the Post-Disaster Setting of Latin America 

**DOI:** 10.3390/ani3030855

**Published:** 2013-08-27

**Authors:** Elena Garde, Gerardo Acosta-Jamett, Barend Mark Bronsvoort

**Affiliations:** 1 Division of Pathway Medicine, School of Biomedical Sciences, College of Medicine and Veterinary Medicine, The University of Edinburgh, Edinburgh, EH16 4SB, UK; 2 Latin America Branch, Veterinarians Without Borders (Veterinarios Sin Fronteras) Canada, Pasaje Los Arrayanes 333, Valdivia, Chile; 3 Instituto de Medicina Preventiva Veterinaria, Facultad de Ciencias Veterinarias, Universidad Austral de Chile, Casilla 567, Valdivia, Chile; E-Mail: gerardo.acosta@uach.cl; 4 The Roslin Institute and Royal (Dick) School of Veterinary Studies, University of Edinburgh, Midlothian, EH25 9RG, UK; E-Mail: mark.bronsvoort@roslin.ed.ac.uk

**Keywords:** disaster, free-roaming dogs, Latin America, preparedness planning, canine zoonosis

## Abstract

**Simple Summary:**

Free-roaming dogs are seldom considered an important public health risk following natural disasters in developing regions. With the high number of recognized canine zoonoses and evidence of increased transmission of some significant diseases this is a risk that may be being overlooked. Communities with free-roaming dogs and endemic canine zoonoses of importance should be developing appropriate community preparedness and response plans to mitigate the occurrence of increased transmission following disasters.

**Abstract:**

In the absence of humane and sustainable control strategies for free-roaming dogs (FRD) and the lack of effective disaster preparedness planning in developing regions of the world, the occurrence of canine zoonoses is a potentially important yet unrecognized issue. The existence of large populations of FRDs in Latin America predisposes communities to a host of public health problems that are all potentially exacerbated following disasters due to social and environmental disturbances. There are hundreds of recognized canine zoonoses but a paucity of recommendations for the mitigation of the risk of emergence following disasters. Although some of the symptoms of diseases most commonly reported in human populations following disasters resemble a host of canine zoonoses, there is little mention in key public health documents of FRDs posing any significant risk. We highlight five neglected canine zoonoses of importance in Latin America, and offer recommendations for pre- and post-disaster preparedness and planning to assist in mitigation of the transmission of canine zoonoses arising from FRDs following disasters.

## 1. Introduction

Domestic dogs have important work and companionship roles in most parts of the world and often cohabitate closely with the human community [1]. In developing countries in Latin America, a vast number of these dogs are free-roaming and are traditionally afforded inadequate veterinary care and husbandry, presenting a highly complex public health problem in which dogs provide a potentially uncontrolled reservoir for zoonoses [1,2]. Although there are more than 300 recognized canine zoonoses [3,4] very little is understood about the changed dynamics that occur between free-roaming dog (FRD) populations, human vulnerabilities and environment following natural disasters that could alter or increase the potential for emergence and transmission of these important diseases in dogs and people. 

In the aftermath of Hurricane Katrina in the United States, dogs were reported as having a number of zoonotic pathogens and recognized as potentially contributing to the geographic expansion of diseases following translocation [5]. Weeks following this high profile disaster, the Centers for Disease Control and Prevention, the American Veterinary Medical Association, American Heartworm Society and the Koret Shelter Medicine Program developed guidelines to control infectious diseases in the post-disaster shelter setting and in 2006 the Pets Evacuation and Transportation Standards Act was approved in the United States requiring the incorporation of pet management into state disaster planning [5,6]

These disaster planning guidelines, however beneficial to North America, are not recognized, nor are they relevant, in developing countries such as in Latin America, where disasters are frequent [7] and the sociocultural realities, local management, resources and infrastructure differ greatly from those seen in developed countries. With less infrastructure and fewer resources to allocate toward dog control, health and surveillance programs [1], effective dog control is rarely achieved [2].

The objective of this article is to suggest the potential for an increase in risk of canine zoonoses following disasters, to highlight five examples of major canine zoonoses of importance in Latin America, and to discuss the importance of developing socially, culturally and economically appropriate disaster preparedness plans for FRDs in Latin America. 

## 2. Disasters and Canine Zoonotic Diseases in Latin America

Natural disasters are common events in the history of Latin America ranking second in the world only to Asia [7]. They cause an estimated 7,500 human deaths per year, have long-term health and economic consequences and are expected to increase in Latin America over time due to climate change [7]. Not surprisingly, developing countries are disproportionately affected, due to their socioeconomic vulnerabilities, and their lack of resources, infrastructure and disaster preparedness [8,9,10]. 

When disasters strike in any part of the world, the ensuing chaos can result in a sudden FRD population caused by owner relocation to shelters and subsequent homelessness and abandonment of pets [11,12]. In Latin American countries, where FRDs are ubiquitous, the associated public health and welfare issues are compounded following disasters. The loss of a regular food source and disruption of established social hierarchies and territories are examples of highly stressful events for dogs, and can lead to decreased immunity, emergence of canine endemic diseases and other negative social behaviors such as fighting and hunting [12]. 

For these reasons, stray dog control is reported as being one of the primary veterinary public health relief actions following a disaster to prevent dog-related hazards such as human-directed bites and attacks, environmental contamination and canine zoonoses [5,11,12,13]. Yet international health agencies responsible for the coordination of disease surveillance in people and livestock are not required to monitor companion animals for emerging or existing zoonoses [1]. Following Hurricane Katrina, researchers detected one or more pathogens of animal or zoonotic importance in the majority of rescued dogs tested, and most had evidence of vector borne diseases [5]. This study highlighted the risks associated with rescuing dogs that could be harboring previous conditions, subclinical infections or of housing high densities of vulnerable animals in poor health, and the potential for expanding the geographic range of diseases through relocation of lost or abandoned dogs.

The most commonly reported human diseases and clinical syndromes following disasters include cholera, bacillary dysentery, viral hepatitis, typhoid fever, gastroenteritis, measles, respiratory viruses, meningitis, malaria, and dengue fever [4,8,10,14]. Under post-disaster circumstances, the cause of disease is seldom confirmed with laboratory diagnosis due to low priority [15] given the concurrent loss of infrastructure, lack of available medical personnel, and the strain on resources. Yet recommendations for conducting epidemiological studies following disasters include laboratory confirmation of disease because not all communicable diseases can be clinically diagnosed based on presenting symptoms and the probability of misdiagnosis increases in the post-disaster scenario where relief medical staff lack the experience in diagnosing local diseases [16]. For example, in Latin America the clinical diagnosis of dengue and typhoid fever are frequently confused [16]. Under normal circumstances, the burden of endemic diseases, many being zoonotic, is grossly underestimated in developing countries and much of this is directly due to poor diagnostic capacity and confusion between diseases [17]. These factors coupled with a low index of suspicion for canine zoonoses could be resulting in a failure to recognize or prevent an increase in transmission of other important diseases. For example in areas highly endemic for malaria, cases of fever are often assumed to be malaria [17] despite the high number of other causative agents that can cause fever, including some serious canine zoonoses such as rabies [18], leptospirosis [19] and leishmaniasis [20]. This practice of unconfirmed diagnosis can lead to a high level of under-reporting of other diseases [17]. 

There are a number of neglected and under-reported canine zoonoses that occur in Latin America that have far-reaching impacts on individuals and communities. These include rabies, leptospirosis, Chagas disease, hydatid disease and leishmaniasis [17] and should be of particular concern following a disaster. These diseases have a broad range of symptoms, affecting multiple systems, and their emergence can follow a disruption in the ecosystem [21] such as that seen following natural disasters. Further complicating the epidemiological picture, some of these diseases such as Chagas disease and hydatid disease are slowly progressive illnesses [22,23] making them difficult to detect and conduct an epidemiological traceback. Here we highlight the pertinent points of these diseases, their relationship to FRDs, and any prior associations with natural disasters for these five canine zoonoses.

### 2.1. Rabies

Rabies has the highest case fatality rate of any known infectious disease but continues to be seriously neglected, under-reported and misdiagnosed in developing countries [1,17,24]. This deadly disease continues to kill between 30,000 and 60,000 people annually worldwide, and dogs are responsible for between 75% and 99% of all human cases in developing countries owing to poor management and low vaccination coverage [1,4,25]. Contrast this with 0.1–5% of human rabies cases in developed regions attributable to dogs purportedly due to confinement and vaccination, and we begin to see the significance of FRD management and healthcare in the perpetuation of this disease [26]. 

In many countries in Latin America, dog-transmitted rabies has been reduced significantly in the last 30 years due to efforts led by Panamerican Health Organization. However, across the region, more efforts are needed, since control strategies are seriously impeded by poverty and the unfavorable sanitary conditions under which many people are living [27]. Natural disasters are reported as one of the factors interfering with the implementation of successful control programs [28].

In 1976, post-earthquake Guatemala City reported an increase in dog bites and there was concern of a rabies outbreak in dogs and people. Fortunately there was no reported increase in human cases [29]. However this example demonstrates a change in FRD dynamics and the inherent risks associated with FRDs in the post-disaster environment (e.g., higher densities, increased aggression, competition and dog-bites, low vaccine rates, and reduced diagnostic capacity in dogs and post-exposure prophylactic treatment in humans) that could increase the risk of a rabies outbreak following disasters. These factors emphasize the need for incorporation of ongoing rabies control and heightened surveillance in the canine population following disasters in countries where the disease is endemic [4,25]. 

### 2.2. Leptospirosis

Leptospirosis is the most common bacterial zoonosis with worldwide distribution, caused by pathogenic spirochete bacteria [30]. Dogs are common carriers of *Leptospira icterohaemorrhagiae* [4] which is the most commonly reported leptospiral infection in humans, and although the natural hosts are rodents, infection in humans is positively associated with exposure to infected dogs [31]. 

Subclinical carriers represent chronic sources of environmental contamination leading to waterborne or foodborne infections or direct infection via contact with people such as petting or licking [4,19,32]. Risk factors for leptospirosis outbreaks in people include poor hygiene and sanitation, crowding, poor waste management, poor rodent control, exposure to infected pets, having free-roaming animals, slaughtering animals, using or bathing in shared water sources, and exposure to contaminated food or water [30] all of which are amplified in post-disaster shelters or camps. Dog foraging activities such as the scattering of garbage and the accumulation of waste in camp areas increases the colonization of rodent hosts [5,33], and behaviors such as male dog urine marking in shelter camps can lead to increased contamination of shared environments. Outbreaks are mostly associated with flooding, but any event, bringing animals and people together, poses a risk for the emergence of leptospirosis [30]. 

Leptospirosis can result in a wide range of symptoms and is commonly misdiagnosed as malaria, hepatitis A and E, influenza, meningitis and pneumonia [30,34], all of which are among the most commonly reported illnesses in people in temporary camps following disasters [14]. For example, following Hurricane Hortense in Puerto Rico 1996, 15 human cases of death were reported as dengue fever but when subsequently autopsied, the actual cause for six of those was found to be leptospirosis [35]. This example offers a specific case of mis-diagnosis following a disaster resulting in the under-reporting of zoonosis. 

### 2.3. Chagas Disease

Chagas disease is the most important parasitic disease, and one of the many canine vector-borne diseases (CVBDs) of the Americas: human infection is directly associated with poverty [17]. Millions of people are infected in Latin America and there are at least 20,000 deaths annually, but it is presumed that the actual prevalence is underestimated [17,35]. 

Dogs plays an important role in human transmission; in fact the triatomine insects responsible for the transmission of *Trypanosoma cruzi* actually prefer the blood of dogs and are 500 times more likely to become infected with the causative protozoon agent when biting an infected dog, than if it bites an infected human [4]. 

In 2002, Hurricane Isidore devastated the Yucatan Peninsula and researchers found that there was a marked increase in the number of triatomine insects found in homes directly along the path of the hurricane. Unfortunately, there was no discussion of the changed dynamics between dogs, people and environment, and due to the slow development of this disease, the new prevalence of Chagas in the local residents remains unknown [35]. 

### 2.4. Hydatid Disease

*Echinococcus granulosus* is a cestode in domestic dogs that causes cystic hydatid disease in humans and livestock: the cycle is perpetuated through the feeding of cyst-bearing offal to dogs following slaughter. The disease is a major public health concern in South America [4,17]. Transmission from dogs to humans is through accidental ingestion of cestode eggs in contaminated food, water or environments or through direct contact with infected dogs [36]. 

In a 2010 study of hydatid disease following the Yushu earthquake in China [37], the investigators suggested that post-disaster conditions may lead to increased transmission of the parasite from dogs to humans. Poor community sanitation resulting in contamination of food and water, increased numbers of roaming dogs, increased access of dogs to raw offal following inappropriate disposal of innards following livestock slaughter, and scavenging of animals that perish in the disaster event were thought to have contributed to an increase in human prevalence in the years following the disaster [37]. As with Chagas disease however, hydatid disease is slowly progressive and requires long term monitoring in order to detect an increase in prevalence due to a particular event [38], while disease studies following disasters typically tend to be short term [29].

### 2.5. Leishmaniasis

Leishmaniasis is another important CVBD. It is the third most important vector-borne disease worldwide and deserves attention in the post-disaster scenario [17]. There is so little known about this disease, yet it is highly prevalent around the world with at least 50,000 deaths annually, an estimated 500,000 new cases annually, is likely seriously under-reported, and is increasing in geographic distribution [1,17,39]. Dogs are the main reservoir hosts for *Leishmania infantum* which is a significant zoonosis in parts of Latin America, particularly in Brazil [40]. Surveillance and control of this disease in the dog is said to be fundamental in controlling the disease in humans [1,40]. Leishmaniasis is endemic throughout South America and an estimated 3,500 cases of canine-transmitted leishmaniasis are reported annually in Brazil alone [39,40]. Although infection in dogs can in many cases remain subclinical, clinical disease when present, affects both humans and dogs, and is often fatal in advanced stages [40]. Signs are not pathognomonic: in fact they are so variable including intermittent fever, diarrhea, lethargy, vomiting, jaundice, cough and skin lesions—most of which can be easily mistaken for other illnesses [4,41,42]. Risk factors are poorly understood, but high numbers of FRDs, any outdoor dogs with exposure to the sand fly vectors, access to forested areas, and those that are young or undernourished are at risk [39,40]. 

Recommendations to prevent infection include the use of screens in homes, and vaccination and insecticidal collars or spot-on treatments for dogs to reduce contact with sand flies but these prevention strategies are difficult to provide in temporary camps following disasters where contact with vectors is almost inevitable [10,43]. Widespread prevention campaigns for pet dogs and FRDs would be challenging to implement in the absence of a coordinated preparedness and response plan [40]. In Brazil, culling of FRDs continues to be the control strategy, despite its inefficacy and lack of acceptance by residents [40]. Immediate diagnosis and rapid initiation of treatment are essential, both of which are complicated [42] and highly unlikely in the post-disaster situation where resources are limited.

## 3. Recommendations and Conclusion

There are numerous canine zoonoses with distribution throughout Latin America and worldwide such as the many CVBDs (eg: *Babesia canis canis*., *Ehrlichia canis, Bartonella nehselae, Borrelia burgdorferi and Rickettsiae reckettsii)*, causing a host of symptoms and diseases in people, and whose dynamics are altered following environmental disturbances [44]. This article highlights five important examples to illustrate the significance of canine zoonoses and to demonstrate the potential for increases in risk following disasters. Factors contributing to the emergence and transmission of these diseases following disasters include the poor level of care and health observed in FRDs, the increased physical contact between people and their dogs in shelters or temporary camps, a poor general understanding of the normal social dynamics and behaviors of FRDs, and a lack of awareness and surveillance for specific canine zoonoses of highest risk. Canine zoonoses causing subclinical infections in dogs, with complex disease expression or challenging diagnoses can be costly and complicated to confirm and therefore control [40]. Those having long incubation periods or those that are slowly progressive present an added impediment because it is difficult to trace diseases back to an original source or time period and detection of increased transmission is complicated. 

Existing recommendations to mitigate the transmission of canine zoonoses following a disaster are restricted to developing countries and focus on animal shelter intake triage, preventive vaccination and parasite control, and shelter biosecurity. These recommendations are not relevant for Latin American countries in which (1) FRDs are an ongoing problem that is further exacerbated by disasters and (2) shelters are rarely used as a means of domestic dog control. 

The absence of a sustainable strategy for managing FRDs often forces public health agencies and municipalities to resort to inhumane methods of removing large numbers of FRDs (e.g., strychnine poisoning) during times of crisis, such as disasters [2,24]. Although mass killing to control dog populations and their diseases may be the only tool available to many managers, it is no longer considered acceptable internationally; yet feasible and humane control strategies remain elusive [2,40]. Alternative strategies for FRD management and disaster planning in Latin American countries must be developed by communities to ensure the inclusion of social, economic, cultural and local realities. Strategies must be regionally relevant, feasible, economically sustainable, and supported by local stakeholders. At minimum, a comprehensive plan should include three major components comprising both pre- and post-disaster planning. 

(1) Management strategies for the control of FRDs. Humane control and management of FRDs in the absence of crisis is a critical step toward reducing the public health and welfare risks following disasters. If FRDs continue to be the norm in Latin American countries, the associated risks that pervade communities will continue to be exacerbated by disasters. Essential components of FRD management should include the development of a written plan by relevant stakeholders and must involve interdisciplinary communications [40,42]. Due to the complexity of the problem and the associated controversies often present, using a participatory and inclusive process is often met with higher success and greater sustainability [45]. Guidance for following a step-wise process and a description of the critical components of FRD management plans have been developed by organizations such as the International Coalition for Animal Management. These include initial assessments of the canine population, and tailored actions addressing education, legislation, registration and identification, sterilization, sheltering and rehoming, euthanasia, and vaccination and parasite control [46]. Regionally significant canine zoonoses must be identified and a plan for targeted disease surveillance implemented. Priority research activities should be identified (e.g., FRD population estimates and monitoring, human attitude and perception surveys toward FRDs, risk analysis for canine zoonosis emergence) to provide important information for the formation of a community plan and to establish baseline information from which to monitor progress. 

(2) Awareness and education plan on canine zoonoses. Following identification of high priority canine zoonoses, an awareness and education campaign should be developed targeting human and animal medical professionals including parasitologists, diagnosticians, and epidemiologists as well as at the political level to ensure inclusion of canine zoonoses into the healthcare and disaster response agendas [42]. The coordination across disciplines is critical in the control and prevention of zoonoses both in the absence of disasters as well as post-disaster. Education campaigns should include the recognition of specific zoonotic diseases in humans, awareness building of common misdiagnoses and underreporting of neglected diseases, risk factors for emergence, control and prevention, and diagnosis.

(3) Guidelines for disaster preparedness and response. Written disaster preparedness and response guides should be developed by responsible agencies in consultation with relevant medical professionals. Although it is beyond the scope of this paper to discuss all the components of a disaster preparedness plan, these guides are essentially designed to identify the local mechanisms and capacities in place to cope with disasters. Guidelines assist in establishing the chain of command and communications plan and detail the steps required to mitigate the damages and risks anticipated during the different stages following disasters (e.g., impact, relief and rehabilitation, long term mitigation and preparedness) [47]. The plan must detail actions to take following disasters to address evacuations, rescue, animal care and safety, sheltering, biosecurity and quarantine, control of disease outbreaks, rehoming, and prevention of canine zoonoses. The process of developing a plan helps to identify areas of need (e.g., financial resources, equipment, supplies, laboratory diagnostics, public education, holding facilities for homeless dogs, *etc.*) and technical capacity and additional training required in order to successfully execute a functional plan to manage, control and mitigate FRD-associated risks following disasters [48]. 

We hope that the opinions presented here will stimulate much needed discussion on the topic of FRD control and management and the exacerbation of the negative public health and welfare effects following disasters. Utilizing a more comprehensive approach to canine zoonoses both before and after natural disasters can aid in improved detection, reporting, prevention, control and treatment. 

## References

[1] Day M.J., Breitschwerdt E., Cleaveland S., Karkare U., Khanna C., Kirpensteijn J., Kuiken T., Lappin M.R., McQuisten J., Mumford E., Myers T., Palatnik-de-Sousa C.B., Rubin C., Takashima G., Thiermenn A. (2012). Surveillance of zoonotic infectious disease transmitted by small companion animals. Emer. Infect. Dis..

[2] Dalla Villa P., Kahn S., Stuardo L., Iannetti L., Di Nardo A., Serpell J.A. (2010). Free-roaming dog control among OIE-member countries. Prev. Vet. Med..

[3] Cleaveland S., Laurenson M.K., Taylor L.H. (2001). Diseases of humans and their domestic mammals: Pathogen characteristics, host range and the risk of emergence. Philos. Trans. R. Soc. Lond. B Biol. Sci..

[4] Macpherson C.N.L., Meslin F.X., Wandeler A.I. Dogs, Zoonoses and Public Health.

[5] Levy J.K., Lappin M.R., Glaser A.L., Birkenheuer A.J., Andersen T.C., Edinboro C.E. (2011). Prevalence of infectious diseases in cats and dogs rescued following Hurricane Katrina. J. Am. Vet. Med. Assoc..

[6] Engelke H.T. (2009). Emergency management during disasters for small animal practitioners. Vet. Clin. North Am..

[7] Charvériat C. (2000). Natural Disasters in Latin America and the Caribbean: An Overview of Risk.

[8] Toole M.J., Noji E. (1997). Communicable diseases and disease control. The Public Health Consequences of Disasters.

[9] Pan American Health Organization (2000). Natural Disasters: Protecting the Public’s Health.

[10] Watson J.T., Gayer M., Connolly M.A. (2007). Epidemics after Natural Disaster. Emerg. Infect. Dis..

[11] Pasquali P., Agrimi U., Borroni R., Busani L., Graziani C., Leonardi M., Poglayen G., Macri A., Mantovani A. (2006). Capacity Building for Surveillance and Control of Zoonotic Disease under Emergency Conditions.

[12] Jackman J., Rowan A, Salem D.J., Rowan A. (2007). Free-roaming dogs in developing countries: The benefits of capture, neuter, and return programs. The State of the Animals IV: 2007.

[13] Garde E.J., Pérez G., Acosta-Jamett G., Bronsvoort B.M. (2013). Characteristics of a Canine Distemper Virus Outbreak in Dichato, Chile Following the February 2010 Earthquake. Animals.

[14] Murthy S., Christian M.D. (2010). Infectious diseases following disasters. Disaster Med. Public Health Prep..

[15] Jafari N., Shahsanai A., Memarzadeh M., Loghmani A. (2011). Prevention of communicable diseases after disaster: A review. J. Res. Med. Sci..

[16] Ippolito-Shepherd J., Hansen R., Schramm D. (1982). Epidemiologic Surveillance after Natural Disaster: A Study Guide.

[17] Maudlin I., Eisler M.C., Welburn S.C. (2009). Neglected and endemic diseases. Philos. Trans. R. Soc. Lond. B Biol. Sci..

[18] Hampson K., Dobson A., Kaare M., Dushoff J., Magoto M., Sindoya E., Cleaveland S. (2008). Rabies Exposures, Post-Exposure Prophylaxis and Deaths in a Region of Endemic Canine Rabies. PLoS Negl. Trop. Dis..

[19] Guerra M.A. (2009). Leptospirosis. J. Am. Vet. Med. Assoc..

[20] Chappuis F., Sundar S., Hailu A., Ghalib H., Rijal S., Peeling R.W., Alvar J., Boelaert M. (2007). Visceral leishmaniasis: What are the needs for diagnosis, treatment and control?. Nat. Rev. Microbiol..

[21] Tabbaa D. (2008). Control of zoonoses in emergency situations: Lessons learned during recent outbreaks (gaps and weaknesses of current zoonoses control programmes). Vet. Ital..

[22] Flores-Chavez M., de Fuentes L., Gárate T., Cañavate C. (2007). Diagnóstico de laboratorio de la enfermedad de Chagas importada. Enferm. Infecc. Microbiol. Clin..

[23] Krokos N., Mihailidou E., Karakatsanis A., Drizis T. (2011). A giant hydatid cyst of the liver. Hellenic J. Surg..

[24] Adedeji A.O., Okonko I.O., Eyarefe O.D., Adedeji O.B., Babalola E.T., Ojezele M.O., Nwanze J.C., Amusan T.A. (2010). An overview of rabies- history, epidemiology, control and possible elimination. Afr. J. Microbiol. Res..

[25] Kaare M., Lembo T., Hampson K., Ernest E., Estes A., Mentzel C., Cleaveland S. (2009). Rabies control in rural Africa: Evaluating strategies for effective domestic dog vaccination. Vaccine.

[26] Macpherson C.N.L. (2005). Human behaviour and the epidemiology of parasitic zoonoses. Int. J. Parasitol..

[27] Schneider M.C., Belotto A., Ade M.P., Hendrickx S., Leanes L.F., de Freitas Rodrigues M.J., Medina G., Correa E. (2007). Current status of human rabies transmitted by dogs in latin america. Cad. Saude Publica.

[28] Vigilato M.A.N., Cosivi O., Knobl T., Clavijo A., Silva H.M.T. (2013). Rabies Update for Latin America and the Caribbean. Emerg. Infect. Dis..

[29] Pan American Health Organization (1982). Experiences with communicable disease following natural disasters in developing countries. Disaster Reports Number 2: Jamaica, St. Vincent, and Dominica.

[30] Lau C.L., Smythe L.D., Craig S.B., Weinstein P. (2010). Climate change, flooding, urbanisation and leptospirosis: fuelling the fire?. Tran. R. Soc. Trop. Med. Hyg..

[31] Heath S.E., Johnson R. (1994). Leptospirosis. J. Am. Vet. Med. Assoc..

[32] Burr P., Lunn K., Yam P. (2009). Current perspectives on canine leptospirosis. In. Pract..

[33] Ivers L.C., Ryan E.T. (2006). Infectious diseases of severe weather related and flood related natural disasters. Curr. Opin. Infect. Dis..

[34] Cruz L.S., Vargas R., Lopes A.A. (2009). Leptospirosis: A worldwide resurgent zoonosis and important cause of acute renal failure and death in developing nations. Ethn. Dis..

[35] Ketai L., Currie B.J., Alva Lopez L.F. (2006). Thoracic radiology of infections emerging after natural disasters. J. Thorac. Imaging.

[36] Moro P., Schantz P.M. (2009). Echinococcosis: A review. Int. J. Infect. Dis..

[37] Wang L.Y., Wu W.P., Li S.Z., Fu Q., Wang Q., Tian T., Yang S.J. (2010). The risk evaluation and response to the spread of hydatid disease after Yushu earthquake in Qinghai Province. Chin. J. Parasitol. Parasit. Dis..

[38] Larrieu E., Frider B., Del Carpio M., Salvitti J.C., Mercapide C., Pereyra R., Costa M., Odriozola M., Pérez A., Cantoni G., Sustercic J. (2000). Portadores asintomáticos de hidatidosis: Epidemiología, diagnóstico y tratamiento. Rev. Panam. Salud.

[39] Dantas-Torres F. (2009). Canine leishmaniasis in South America. Parasit. Vectors.

[40] Otranto D., Dantas-Torres F. (2013). The prevention of canine leishmaniasis and its impact on public health. Trends Parasitol..

[41] Ashford R.W. (2000). The leishmaniases as emerging and reemerging zoonoses. Int. J. Parasitol..

[42] Otranto D., Dantas-Torres F., Breitschwerdt E.B. (2009). Managing canine vector-borne diseases of zoonotic concern: Part two. Trends Parasitol..

[43] Lehman J.A., Hinckley A.F., Kniss K.L., Nasci R., Smith T.L., Campbell G.L., Hayes E.B. (2007). Effect of Hurricane Katrina on arboviral disease transmission. Emerg. Infect. Dis..

[44] Otranto D., Dantas-Torres F., Breitschwerdt E.B. (2009). Managing canine vector-borne diseases of zoonotic concern: Part one. Trends Parasitol..

[45] Wates N. (2002). The Community Planning Handbook.

[46] International Companion Animal Management Coalition. www.icam-coalition.org.

[47] Thapliyal B.K. (2003). Training Module on Planning for Disaster Preparedness and Mitigation.

[48] FAO (1998). The Emergency Sequence: What FAO does—How FAO does it: Phase One: Prevention.

